# Mapping variation of extracellular matrix in human keloid scar by label-free multiphoton imaging and machine learning

**DOI:** 10.1117/1.JBO.28.4.045001

**Published:** 2023-04-08

**Authors:** Jia Meng, Guangxing Wang, Lingxi Zhou, Shenyi Jiang, Shuhao Qian, Lingmei Chen, Chuncheng Wang, Rushan Jiang, Chen Yang, Bo Niu, Yijie Liu, Zhihua Ding, Shuangmu Zhuo, Zhiyi Liu

**Affiliations:** a Zhejiang University, College of Optical Science and Engineering, International Research Center for Advanced Photonics, State Key Laboratory of Modern Optical Instrumentation, Hangzhou, China; b Xiamen University, School of Public Health, Center for Molecular Imaging and Translational Medicine, State Key Laboratory of Molecular Vaccinology and Molecular Diagnostics, Xiamen, China; c Jimei University, School of Science, Xiamen, China; d Zhejiang University, Jiaxing Research Institute, Intelligent Optics and Photonics Research Center, Jiaxing, China

**Keywords:** morphological feature, textural feature, machine learning, classification, keloid scar, multiphoton imaging

## Abstract

**Significance:**

Rapid diagnosis and analysis of human keloid scar tissues in an automated manner are essential for understanding pathogenesis and formulating treatment solutions.

**Aim:**

Our aim is to resolve the features of the extracellular matrix in human keloid scar tissues automatically for accurate diagnosis with the aid of machine learning.

**Approach:**

Multiphoton microscopy was utilized to acquire images of collagen and elastin fibers. Morphological features, histogram, and gray-level co-occurrence matrix-based texture features were obtained to produce a total of 28 features. The minimum redundancy maximum relevancy feature selection approach was implemented to rank these features and establish feature subsets, each of which was employed to build a machine learning model through the tree-based pipeline optimization tool (TPOT).

**Results:**

The feature importance ranking was obtained, and 28 feature subsets were acquired by incremental feature selection. The subset with the top 23 features was identified as the most accurate. Then stochastic gradient descent classifier optimized by the TPOT was generated with an accuracy of 96.15% in classifying normal, scar, and adjacent tissues. The area under curve of the classification results (scar versus normal and adjacent, normal versus scar and adjacent, and adjacent versus normal and scar) was 1.0, 1.0, and 0.99, respectively.

**Conclusions:**

The proposed approach has great potential for future dermatological clinical diagnosis and analysis and holds promise for the development of computer-aided systems to assist dermatologists in diagnosis and treatment.

## 1 Introduction

Excessive scarring is mainly caused by the abnormal healing of physiological wounds, including burn injuries, lacerations, abrasions, surgeries, and vaccinations. It can dramatically affect patients’ quality of life by causing pruritus, pain, and contractures.^1^^–^^4^ In particular, keloid scarring is unique to humans.^5^ No other animal species have been found to naturally develop scar tissue compared to that of human keloids.^6^ In addition, keloids continue to grow and extend beyond the original wound margins.^7^^,^^8^ Notably, sporadic case reports show that keloids may be a paraneoplastic phenomenon.^9^^–^^13^ Keloid scar could develop within years after initial injury, and some of them may persist for a lifetime without treatment. The etiology of keloid scar formation is not completely understood, and the recurrence rate of this disease is relatively high.^14^^,^^15^ Thus the identification and evaluation of keloid tissues and their boundaries, especially at relatively early stages, are the most critical steps in determining the degree of tissue damage and appropriate treatment for scar management and removal. In this context, it is essential to gain a thorough understanding of the microstructure in the extracellular matrix (ECM) of normal, keloid, and adjacent regions and to accurately distinguish them.

Generally, if a keloid looks like a worrisome skin growth, a skin biopsy must be performed. To improve tissue contrast and highlight characteristics for analysis, several histological dyes, including HE,^16^ picrosirius red,^17^ and Masson’s trichrome,^18^ have been utilized to stain tissue components. However, the cumbersome procedure, including tissue fixation, embedding, sectioning, and staining, requires a considerable waiting time. Furthermore, the histological image interpretation by dermatologists may introduce interobserver bias, which could affect the accuracy of diagnosis. Hence, it is necessary to design a rapid, nondestructive, label-free, and accurate method to improve the efficiency and accuracy of keloid diagnosis and analysis.

Multiphoton microscopy based on second harmonic generation (SHG) and two-photon excited fluorescence (TPEF) allows specialists to perform label-free detection of tissues (e.g., collagen, elastin, muscles, and cells). It also allows for nondestructive assessments by preserving the original state of the tissue without destructive treatment, such as ionization, which is typically required by technologies including electron microscopy and liquid chromatography–mass spectrometry.^19^^,^^20^ In addition, alterations in morphology and organization of collagen and elastin fibers, the major components of the ECM, are closely associated with physiologic and pathologic status of biological tissues.^21^^–^^25^ Compared with traditional pathological methods, multiphoton imaging technology has great benefits in simplifying sample preparation. In the pathological method, it requires fixing the tissue sections with paraffin and staining them. These processes are relatively complicated and time consuming. In contrast, tissues can be imaged directly or after being sectioned, without other special processing. SHG acquires images of collagen fibers without exogenous labeling agents owing to their noncentrosymmetric structure, and elastin fibers are natural fluorophores and provide TPEF signals.^26^ Therefore, multiphoton imaging technology leads to label-free imaging of these two main components within the ECM. In addition, it has a high resolution at the submicron level and holds the potential of *in vivo* imaging with the development of articulated arm-based detection approaches.^27^ Hence, multiphoton imaging may be suitable for discrimination and analysis of keloid scar, adjacent, and normal regions of skin tissues.

Although the multiphoton imaging is promising for scar imaging, the analysis of multiphoton images by experts is laborious and susceptible to interobserver bias. Computer-aided analysis has been applied to the study of scars. Maknuna et al.^28^ reported the employment of a neural network to identify the scar regions of pathological sections and then analyzed two morphological features (density and alignment) of collagen fibers within scar tissues. The mean average precision was <70%. Only two features might not be sufficient for describing collagen characteristics comprehensively. Moreover, the detection of the scar region (based on deep learning) was separate from the analysis of collagen features, which made it difficult to evaluate the contribution of characteristics to the detection. Wang et al.^29^ proposed a texture-based approach to explore the mechanism of the scar. Generative adversarial network textural analysis was utilized to predict the evolution of the scar, and only six textural features were extracted. These previous studies mainly focused on analysis of collagen fibers, and scar boundary regions were neglected. Furthermore, there was a lack of insightful and fully automated analysis for scar tissues. Therefore, a fully automated approach that provides a comprehensive understanding of the keloid scar by gathering information from both collagen and elastin fibers and accurately identifies the scar boundary region is highly needed.

In this study, we develop a computer-aided diagnosis and analysis platform, which involves a multiphoton imaging module (for both collagen and elastin fibers), a feature selection module [based on minimum redundancy maximum relevancy (MRMR)], and an analysis module [based on stochastic gradient descent and the tree-based pipeline optimization tool (TPOT)] for rapid, automated, and accurate diagnosis and analysis of excised human keloid scar, normal, and adjacent tissues.

## 2 Materials and Methods

### 2.1 Study Design

**Fig. 1:**
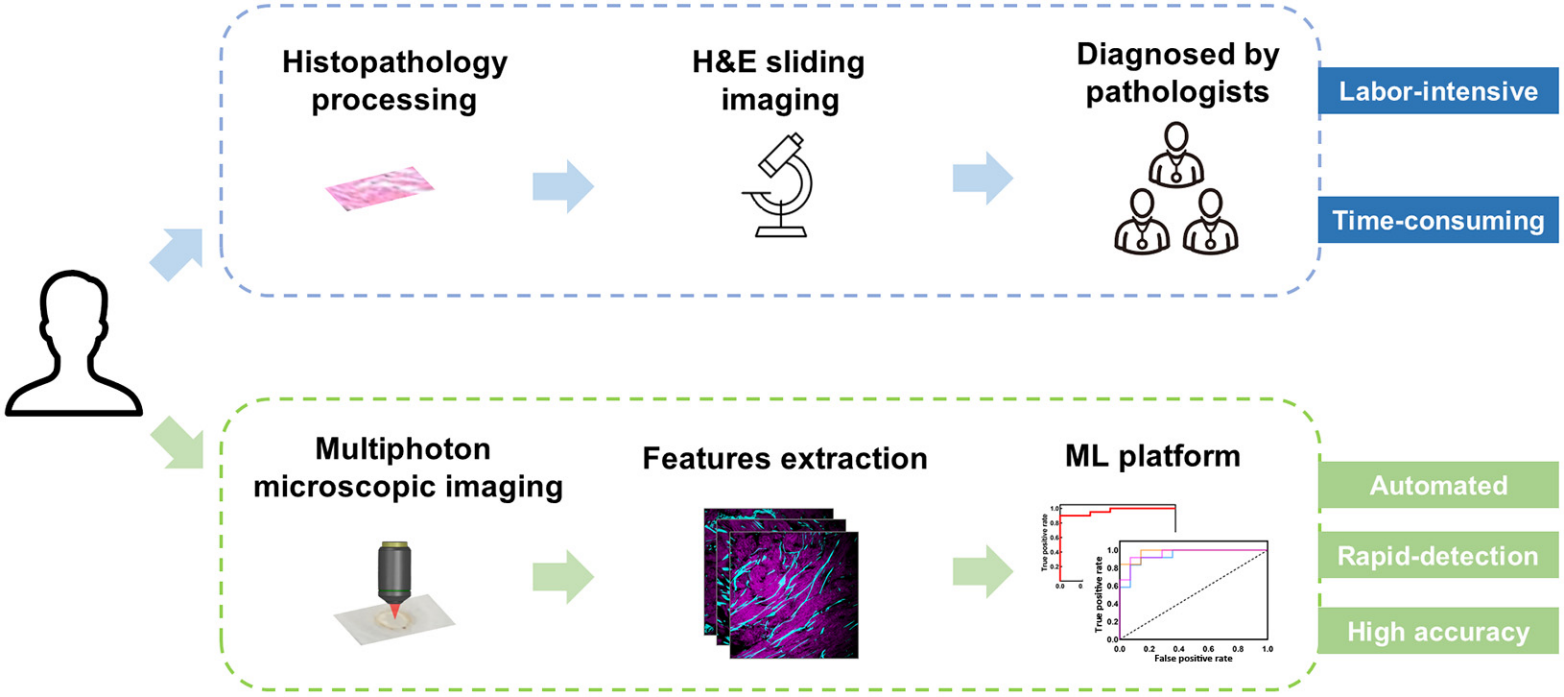
Comparison of traditional pathology methods and the proposed automated methods. Instead of traditional histopathological examination, the excised tissue are instantly imaged without cumbersome procedures. Images are then analyzed in real time with computer assistance.

**Fig. 2:**
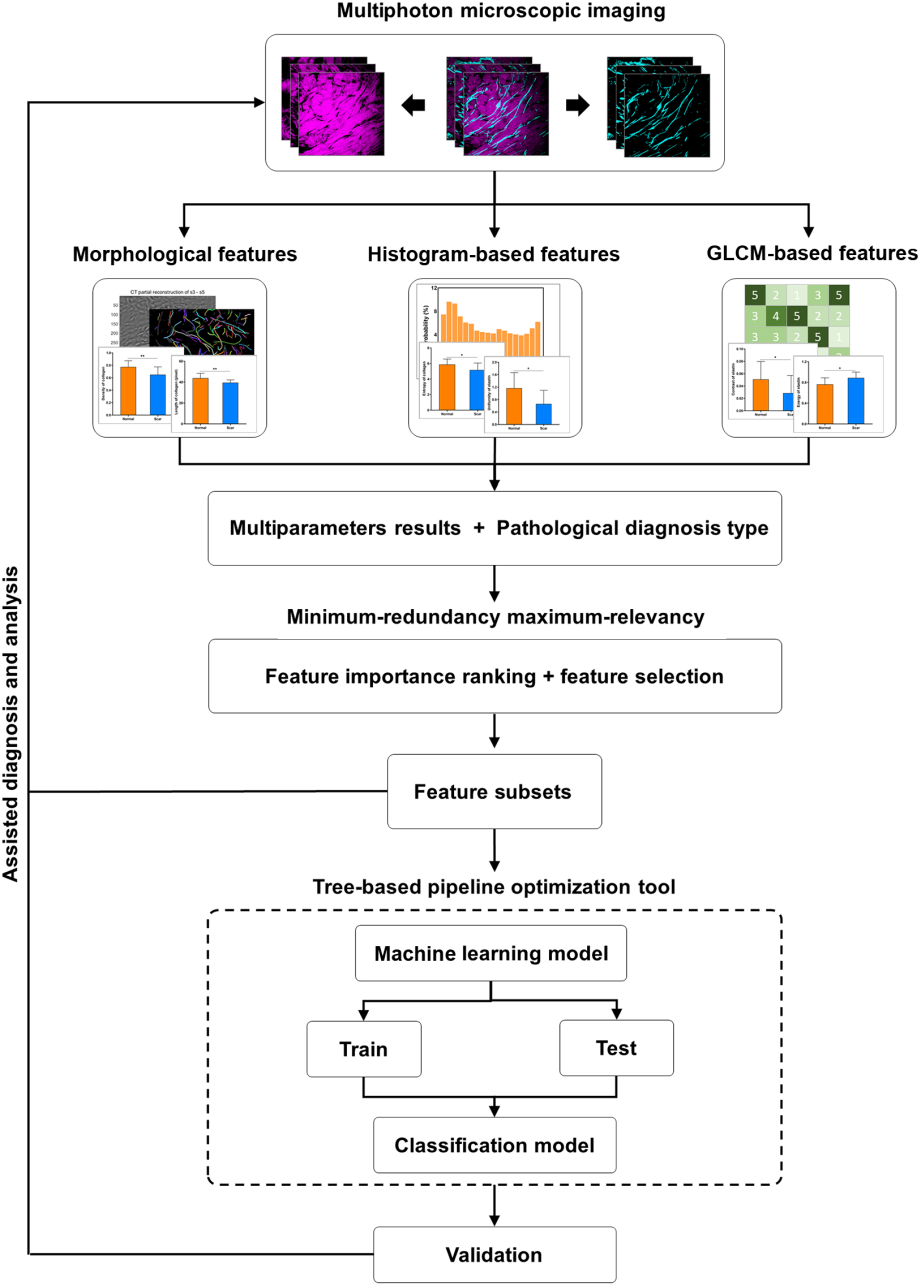
Workflow of the automated diagnosis and analysis system. First, SHG and TPEF images were acquired from human keloid scar tissues. Second, morphological, histogram-based, and GLCM-based features were extracted to quantify the differences among normal, adjacent, and scar tissues. Third, a classifier was developed based on MRMR combined with the TPOT. Feature subsets facilitate deeper analysis of endogenous changes in keloid scar tissues, whereas the classifier model allows for automated diagnosis.

For keloid scars, traditional pathology methods are labor intensive and time consuming, and a more accurate and automated diagnostic system is urgently needed (Fig. 1). An automated classification and analysis system was developed to identify and quantify keloid, adjacent, and normal skin tissues (Fig. 2). The original SHG and TPEF images were first denoised. Then 28 features (14 for collagen and 14 for elastin fibers) were extracted; these included morphological and textural [histogram-based and gray-level co-occurrence matrix (GLCM)-based] feature. Morphological features (8 parameters) were directly related to human visual percept and were widely used in clinical diagnosis. Histogram-based textural features (12 parameters) reflected the texture characteristics of the whole image, without considering information from the relative positions of pixels. GLCM-based textural features (8 parameters), based on the analysis of the co-occurrence matrix, could well reflect the information among neighboring pixels. These were typical characteristics in the field and provided complementary insights into the morphology and organization of both fiber components. All of the features were implemented for importance scoring based on the MRMR method. The TPOT was then used to generate the optimal classifier based on the feature subsets. Feature analysis and classification results could be achieved to assist in the diagnosis and analysis of the keloid scar.

### 2.2 Sample Preparation

The *ex vivo* human specimens were collected from 23 hospitalized patients (aged 30 to 58 years old, scar duration: 0.5 to 2 years). Informed consent was obtained from each patient who participated in this study. The ethics approval and experimental procedures were approved by the Institutional Review Board of Fujian Medical University. After excision from patients, the tissue samples were quickly frozen and stored in liquid nitrogen (−196°C). For multiphoton imaging, the tissue sections were cut into 30-μm thickness and sandwiched between a microscope slide and cover glass. In this study, we imaged and analyzed 26 tissue section samples (16 scar ones and 10 normal ones). From eight different scar specimens, we obtained the adjacent regions. The imaged regions were marked after the imaging procedure and then prepared for the pathological staining. Tissue types (normal, scar, or adjacent) of the imaged regions were confirmed by experienced pathologists according to the staining readouts. To avoid dehydration or shrinkage during imaging, a small amount of phosphate buffered saline solution was dropped into the sample.

### 2.3 Multiphoton Imaging System

A nonlinear optical imaging system was built using a commercial laser scanning microscope (Zeiss, LSM 510) and mode-locked Ti:Sapphire fs laser (110 fs, 76 MHz), which was tunable from 700 to 980 nm. The laser beam was scanned in the focal plane by a galvanometer driven optical scanner. A dichroic beam splitter was applied to reflect the excitation laser and direct the fluorescent and SHG signals to the detector. A large aperture, oil immersion objective (63×, NA=1.4) was employed for high-resolution imaging. The SHG and TPEF signals were collected by the detector, which covered a spectral window of 340 nm ranging from 377 to 716 nm. Two different channels were selected to obtain the images of collagen and elastin fibers. SHG signals were obtained using the channel with the wavelength ranging from 398 to 409 nm, whereas TPEF signals were collected by the channel covering the wavelength range from 430 to 697 nm. Images were taken with a field of view of 210  μm×210  μm and an acquisition time of 680 ms per image. We imaged two or three neighboring regions from each tissue section (i.e., 16 scar ones and 10 normal ones) for the subsequent analysis. In addition, we obtained images from scar-normal adjacent regions from eight independent scar specimens. Characteristic features from multiple regions of each sample were then averaged as outputs.

### 2.4 Features Extraction

#### 2.4.1 Morphological analysis

The morphological analysis of collagen and elastin fibers included density, alignment, width, and length (detailed in Table S1 in the Supplementary Material). Density is the percentage of effective pixels over the total number of pixels. The quantification of the alignment of the scar tissues was performed using the fast Fourier transform and semicircular von Mises distribution calculated by FiberFit, which provided the alignment assessment by parameter k, with smaller k values indicating more disordered fibers and larger values corresponding to a better alignment.^30^

The fiber width and length were quantified using the curvelet-transform fiber-extraction algorithm (CT-FIRE), which has been successfully used to characterize fiber morphology in several diseases.^31^ This method combined the advantages of the fast discrete curvelet transform for denoising images and the fiber extraction algorithm for extracting fibers.^32^

#### 2.4.2 Texture analysis

To obtain the texture characteristics of an image, statistical properties of the intensity histogram were assessed (Table S1 in the Supplementary Material). The mean intensity, standard deviation, smoothness, skewness, uniformity, and entropy were calculated from the histogram of the SHG and TPEF pixel intensity distributions. Using histogram only without information from relative position of pixels, herein, we also acquired GLCM-based texture features (Table S1 in the Supplementary Material), including contrast, correlation, energy, and homogeneity (with descriptions and calculation methods detailed in the Supplementary Material).

### 2.5 Feature Selection

The MRMR feature selection approach^33^ was employed as the filtering method in the classifier construction to improve the performance of the classification model. This approach found the most relevant features based on their correlation with the target and to reduce the redundancy of the extracted features, revealing the features that had maximum relevancy and minimum redundancy. Therefore, feature importance ranking, that is, the ability of a feature to distinguish different groups of samples, was obtained accordingly. In terms of information theory, mutual information is defined as

$$I(x;y)=\iint p(x,y)\;\log\frac{p(x,y)}{p(x)p(y)}dx\;dy,$$

where I(x;y) is the mutual information between two random features x and y and p(x), p(y), and p(x,y) are the probability density functions of x and y. The purpose of feature selection is to find a feature set S with m features that had the largest dependency on the target c:

$$\max\;D(S,c),D=\frac{1}{\left|S\right|}\underset{x_{i}\in S}{\sum }I(x_{i};c).$$

To eliminate redundancy among features, the minimum redundancy criterion was used and is defined as

$$\min\;R(S),\;R=\frac{1}{|S{|}^{2}}\underset{x_{i},x_{j}\in S}{\sum }I(x_{i};x_{j}).$$

The incremental search methods can be utilized to find the near-optimal features. Suppose that we had $S_{m-1}$, the feature set with m−1 features, and the task was to select the m’th feature from the set $\left\{X-S_{m-1}\right\}$. This feature was chosen by maximizing the single-variable relevance minus redundancy function, which is calculated as

$$\underset{x_{j}\in X-S_{m-1}}{\max}[I(x_{j};c)-\frac{1}{m-1}\underset{x_{i}\in S_{m-1}}{\sum }I(x_{j};x_{i})].$$

### 2.6 Statistical Analysis

A two-tailed Mann–Whitney test was applied to determine significant differences among the mean values from different groups (p<0.05 was considered to be significantly different). We utilized machine learning to build a classification model based on the extracted features. Here stochastic gradient descent classifier was adopted to discriminate normal, scar, and adjacent tissues. To optimize the classifier performance, the TPOT^34^—a genetic programming-based machine learning tool—was used. The genetic programming settings used in this study are listed in Table 1. Considering the relatively small number of samples, we implemented the leave-one-out (LOO) method for cross validation. Each sample was used once as a test set, and the remaining samples formed the training set. Compared with other methods, such as k-fold cross validation, this method estimated the model performance critically and made full use of all data, and the results were deterministic (repeatable). When the datasets were large, the computation time cost of this method would be huge. Therefore, the LOO method was especially suitable for small datasets.

**Table 1:** Genetic programming parameters for the TPOT.

Parameter	Value
Population size	50
Generations	50
Fitness function	$\text{Accuracy}=\frac{1}{n_{\text{samples}}}\overset{n_{\text{samples}}-1}{\underset{i=0}{\sum }}l({\text{value}}_{\text{predicct}}={\text{value}}_{\text{true}})$
Selection	10% elitism, rest three-way competition
Crossover	One-point crossover
Mutation rate	90%
Crossover rate	10%

## 3 Results

**Fig. 3:**
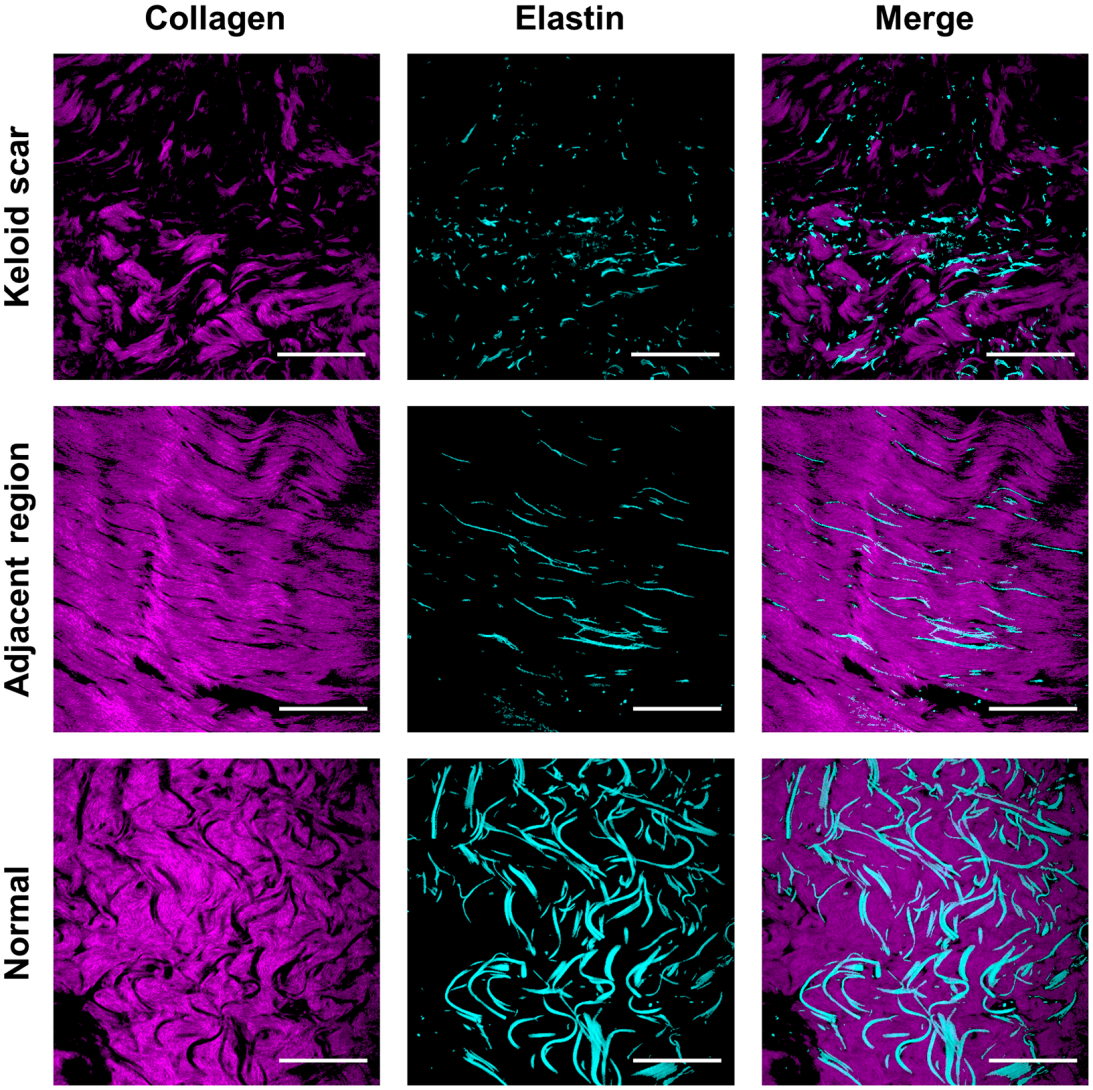
Representative collagen (obtained from SHG) and elastin (obtained from TPEF) images of human skin tissue specimens, including normal, scar, and adjacent regions. Scale bar: 50  μm.

Figure 3 shows representative examples of different tissue types. Normal skin tissue was characterized by intact and continuous collagen and elastin fibers. In keloid scar tissue, the fibers were discrete and more disordered. Interestingly, adjacent tissues showed a highly ordered arrangement of collagen and elastin fibers.

**Fig. 4:**
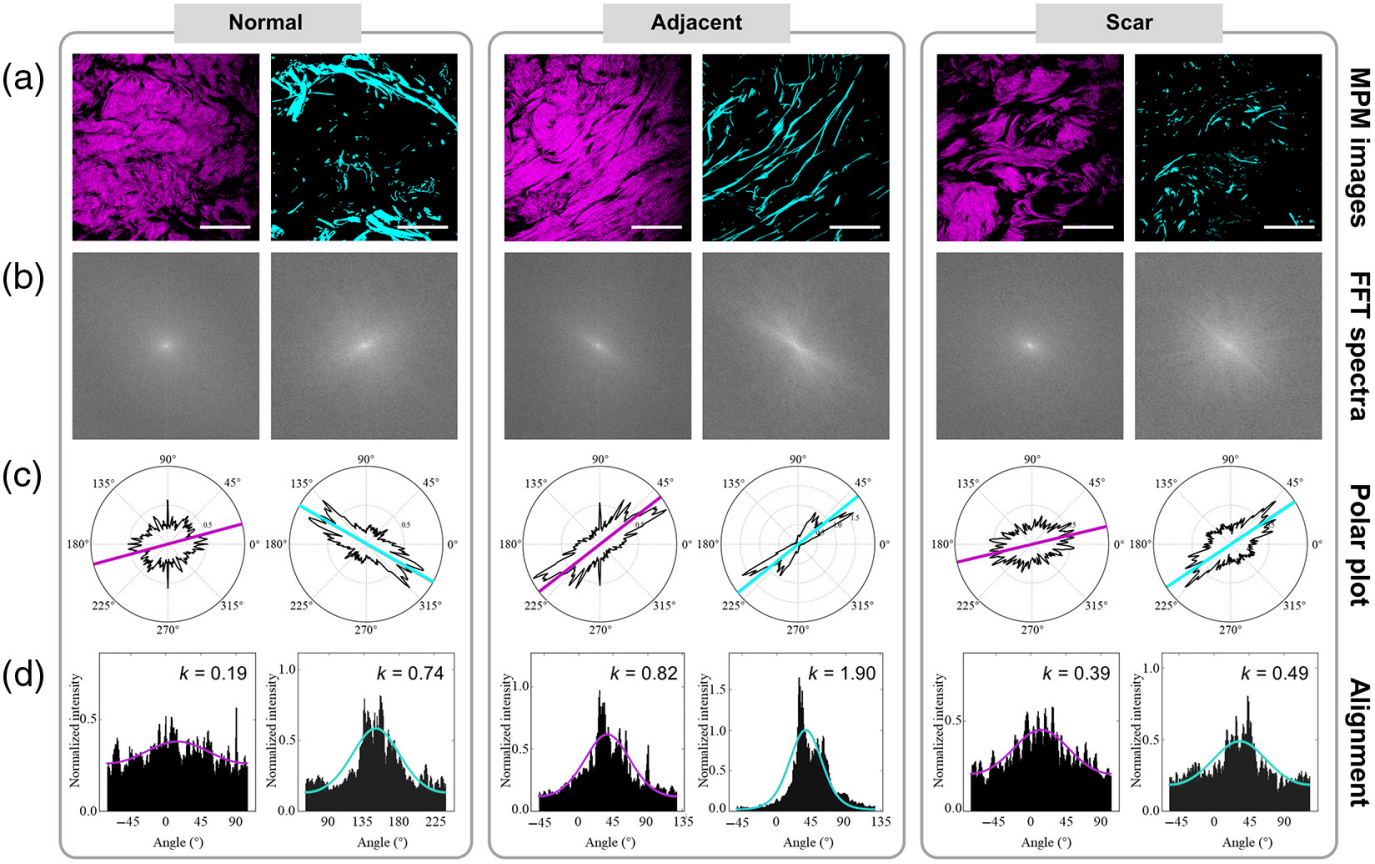
Analysis of collagen and elastin alignment: (a) representative images, (b) FFT spectra, (c) polar plot, and (d) orientation distributions of collagen and elastin for normal, adjacent, and scar tissues. Scale bar: 50  μm.

The fast Fourier transform and semicircular von Mises distribution method was used to characterize the alignment of the collagen and elastin fibers in these tissues. Figure 4 shows the quantification results for representative collagen and elastin fibers. The FFT spectra [Fig. 4(b)] of the SHG and TPEF images [Fig. 4(a)] were obtained to reflect the image intensity characteristics. A radial sum approach was utilized to create fiber orientation distribution, as shown in Fig. 4(c). The alignment of the collagen and elastin fibers was calculated by fitting the fiber orientation distribution data to a semicircular bon Mises distribution [Fig. 4(d)]. In addition, CT-FIER was implemented to calculate the width and length of the collagen and elastin fibers. Similarly, texture analysis results of images were also obtained.

**Fig. 5:**
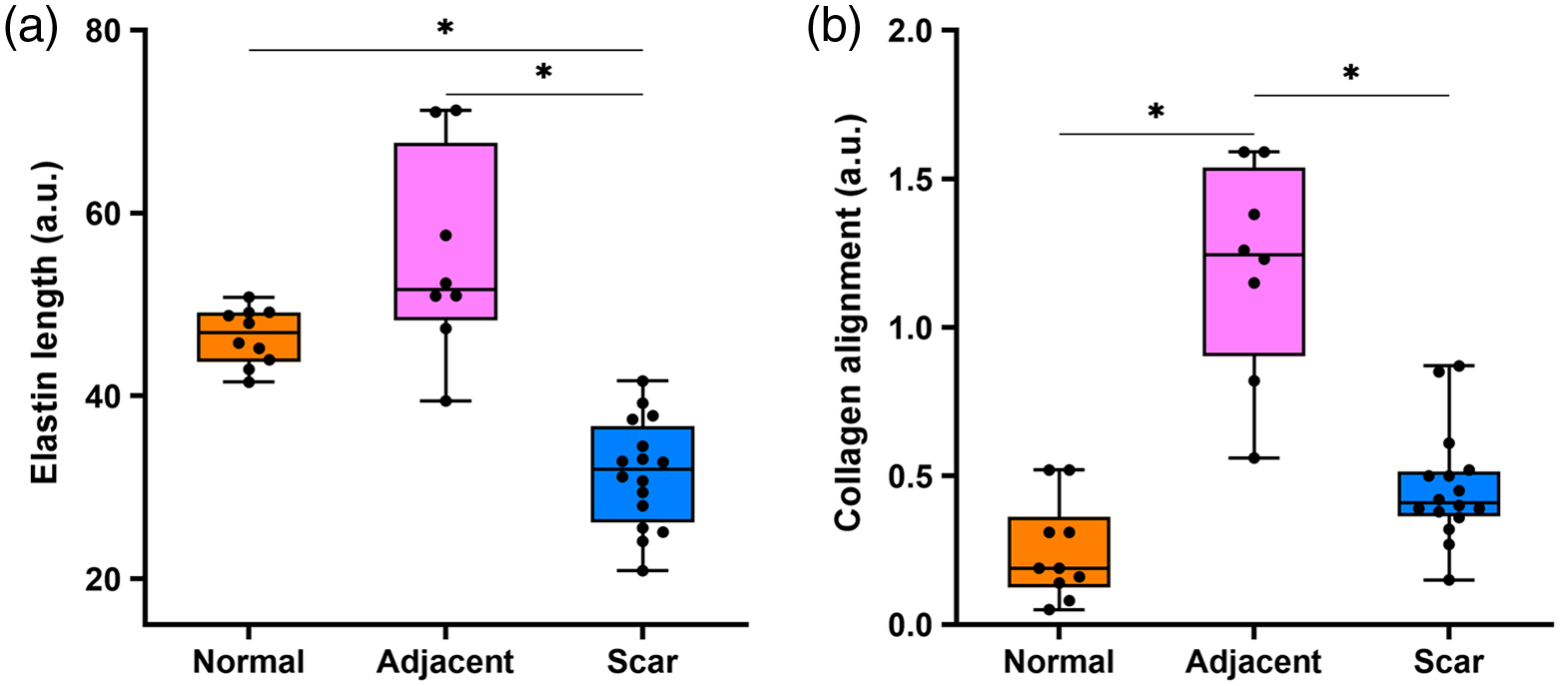
Performance of representative features in distinguishing different tissue types. (a) Boxplot acquired from (a) elastin length and (b) collagen alignment. *p<0.05.

Following 28 features extracted from the collagen and elastin images, we employed the MRMR method to assess the importance of these features. Table 2 shows the feature importance ranking results. Elastin length and collagen alignment were found to be the top 2 ranked important characteristic features, with the corresponding boxplots shown in Fig. 5. Some other representative features for distinguishing different groups are shown in Figs. S1–S3 in the Supplementary Material.

**Table 2:** Rank of features extracted from SHG and TPEF images using the MRMR method.

Feature	Rank	Feature	Rank	Feature	Rank	Feature	Rank
C-density	23	C-skewness	11	E-density	16	E-skewness	19
C-length	6	C-uniformity	20	E-length	1	E-uniformity	14
C-width	13	C-entropy	12	E-width	10	E-entropy	5
C-alignment	2	C-contrast	28	E-alignment	4	E-contrast	25
C-mean Int	8	C-correlation	21	E-mean Int	3	E-correlation	22
C-std	9	C-energy	18	E-std	7	E-energy	27
C-smoothness	26	C-homogeneity	24	E-smoothness	15	E-homogeneity	17

Abbreviations: [C-feature name], feature of collagen; [E-feature name], feature of elastin; mean Int, mean intensity; and std, standard deviation.

**Fig. 6:**
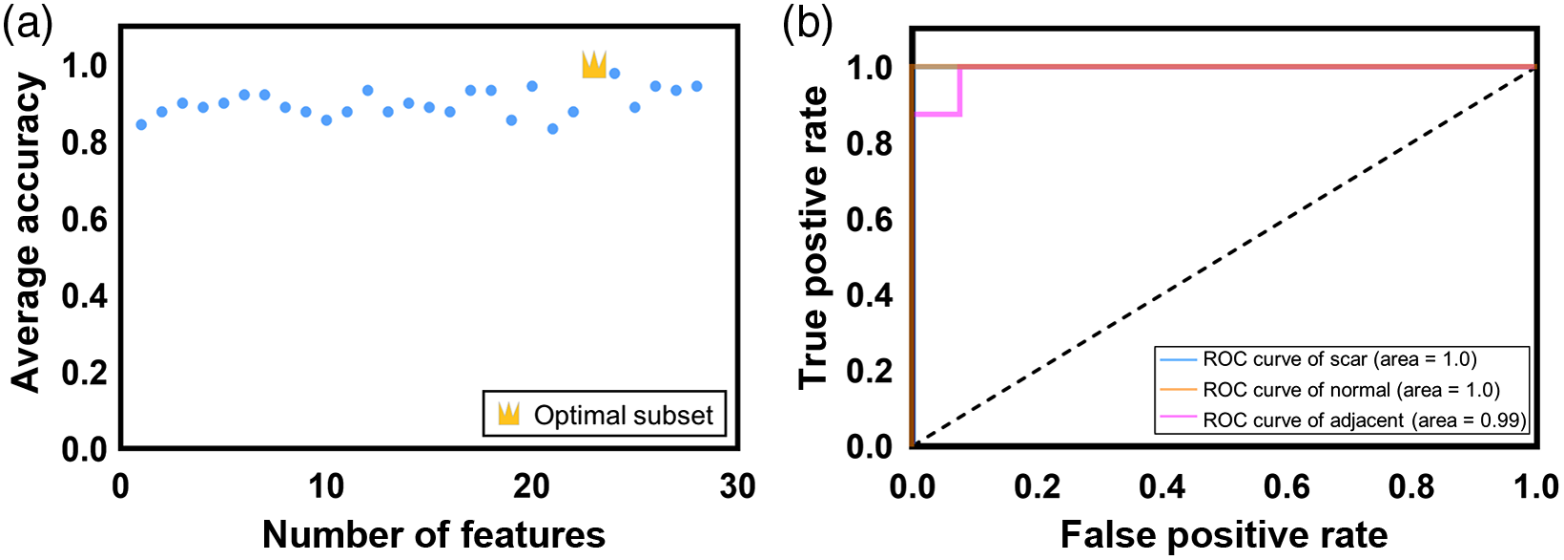
Statistical analysis results: (a) scattering plot of the average accuracy for each feature subset. The best average accuracy is highlighted by the golden crown and is near 1.0 when the top ranked 23 features are utilized. (b) ROC curve and AUC for the classification results, including keloid versus normal and adjacent tissues (blue line), normal versus keloid and adjacent tissues (orange line), and adjacent versus keloid and normal tissues (magenta line). The corresponding AUC values are 1.0, 1.0, and 0.99, respectively.

To determine the feature subsets to obtain the most optimal classifier, the incremental feature selection approach was then implemented. According to the feature importance ranking results, 28 individual feature subsets, with the n’th subset containing n features from the first ranked one, were built to classify different types of tissues. These feature subsets were imported into the TPOT for processing to obtain the optimized machine learning pipeline and the corresponding accuracy. Each of the 28 feature subsets was repeatedly processed five times to compare the average accuracy outcomes. The average accuracy was 71.4% when the most relevant feature, elastin length, was used as a feature subset. The best average accuracy was 100% when the top ranked 23 features were imported into the TPOT, as shown in Fig. 6(a). The TPOT method could obtain the optimized classifier and the corresponding hyperparameters by the genetic algorithm to construct the most suitable machine learning model.

The final machine learning model was the stochastic gradient descent (SGD) classifier optimized by the genetic algorithm. The corresponding hyperparameters of the classifier that were used in the model are listed in Table 3. We performed cross validation of the model using the LOO method, with an accuracy of 96.15% in classifying the normal, adjacent, and keloid scar tissues. The receiver operating characteristic curve (ROC) and area under the curve (AUC) were generated to evaluate the accuracy of the model [Fig. 6(b)]. The AUC values of corresponding classification results (scar versus normal and adjacent, normal versus scar and adjacent, and adjacent versus normal and scar) were 1.0, 1.0, and 0.99, respectively. These results indicated that the proposed model can effectively distinguish normal, scar, and adjacent tissues.

**Table 3:** Hyperparameters of the corresponding optimized classifier.

Classifier	Hyperparameter	Value
SGD classifier	Alpha	0.001
	Eta	0.01
	Fit intercept	True
	Learning rate	Constant
	Loss	Modified huber
	Penalty	elasticnet
	Power t	0.5

## 4 Discussion

Keloid scars are unique to humans. Lesion areas do not regress or contract and might continue to extend beyond the original wound.^6^ Considering the invasiveness of a keloid scar to the surrounding skin, it was necessary to characterize and distinguish the scar, normal, and adjacent regions. Currently, the clinical method for diagnosing keloid was pathological examination, which was time consuming and strenuous. In addition, observation bias might happen due to pathological diagnosis by specialists. Hence, automated, rapid, and accurate diagnosis is highly demanded. We proposed a computer-aided diagnosis and analysis method based on multiphoton imaging and machine learning. This method analyzed skin tissues without complex processing and staining. In addition, scar, normal, and adjacent regions were distinguished by a machine learning model in an automated manner, avoiding observer bias.

Many computer-aided analysis methods for scars had shortcomings in feature extraction and analysis. Collagen and elastin fibers, as important components of the ECM, are closely associated with keloid scar progression. In some studies, elastin fibers were often neglected. However, recently the degradation and disorder of elastin fibers were found to be an important signature during the formation and development of keloid scars.^35^ The importance of elastin was highlighted in our proposed feature importance ranking, with the elastin length being the most powerful parameter in identifying certain tissue types. Meanwhile, adjacent regions played an important role in determining the treatment scope and studying the development of keloid.^36^ By combining morphological and texture features, and usage of MRMR as the feature selection method, we successfully identified adjacent regions with a high accuracy through machine learning.

Our proposed method assisted in keloid scar and boundary diagnosis according to the analysis results and explored the potential pathological features. Initial filtering of features was achieved by the MRMR method. In the triple classification problem, elastin length, collagen alignment, and elastin content (as represented by elastin signal intensity) were found to be sensitive characteristic features, consistent with previous findings.^5^^,^^35^^,^^37^^,^^38^ Elastic fibers in the scar region exhibited lower k values and smaller length values compared with ones in the normal region, reflecting the disruption and disorganization of elastin fibers with scar formation.

The evaluation and treatment of keloid scars are closely related to the health of patients. The most common methods for evaluation relied on the use of a scoring scale (such as Vancouver scar scale) and 2D photography.^39^ However, they were subjective assessments and might affect the accuracy of boundary identification and thus the subsequent treatment. There were various treatment options for keloid scars, including scar freezing, laser treatment, radiation therapy, and surgical removal. Unfortunately, there was no single medical therapy that was shown to be effective in treating keloid scars, and surgical excision was considered ineffective as a monotherapy given the recurrence rate of 45% to 100%,^14^^,^^40^^,^^41^ partly owing to inappropriate determination of the treatment area. To reduce the risk of recurrence, the combination of the surgical removal and conservative treatment method (such as radiotherapy or cryotherapy) was recommended. In this context, the proposed method might help in enhancing the knowledge of this disease and determining the proper treatment regions.

There are some limitations associated with our study. A larger number and age range of patients from multiple hospitals are needed to confirm our method before it is implemented in clinical application. Default parameters of the TPOT were used in our study, whereas some other hyperparameters, including generations and population size, will be considered in our future study. Moreover, the parameters of the genetic algorithm will be analyzed in detail to further improve the model accuracy and analytical capabilities. Our work presents a preliminary investigation, and further studies are required to enable translation of the findings into clinical applications, such as the development of articulated arm-based detection approaches for *in vivo* scar imaging.^27^ In this study, we focused on the diagnosis and analysis of keloid scars, including identifying different regions and extracting important features. It is also important to distinguish keloid tissues from other diseases and skin conditions, which will be a focus in our future work.

## 5 Conclusion

In summary, we visualized the alteration of collagen and elastin organization in scar, normal, and adjacent human skin tissues *ex vivo* by multiphoton microscopy. Morphological features and texture features were extracted from SHG and TPEF images. The extreme gradient boosting method was employed to quantify the importance of features. As a result, the AUC values of classification results (scar versus normal and adjacent, normal versus scar and adjacent, and adjacent versus normal and scar) were obtained as 1.0, 1.0, and 0.99, respectively. Compared with conventional skin biopsy methods, the proposed method allows for automated, rapid, and label-free characterization and diagnosis of keloid scars. In particular, the accurate identification of scar boundary is highly promising. This approach has great potential for future dermatological clinical applications and holds promise for the development of computer-aided systems to assist dermatologists in diagnosis and treatment.
