# Supplementary material for: Mapping variation of extracellular matrix in human keloid scar by label-free multiphoton imaging and machine learning
Source: J Biomed Opt. 2023 Apr 8;28(4):045001. doi: 10.1117/1.JBO.28.4.045001 (PMC10082605; doi:10.1117/1.JBO.28.4.045001)
Supplement: Supplementary file 1 [file JBO_028_045001_SD001.docx]

**Mapping variation of extracellular matrix in human keloid scar by label-free multiphoton imaging and machine learning**

**1. Supplementary Methods**

- 1. *Histogram-based texture analysis*

To quantify intensity features, a histogram-based approach was utilized. The mean intensity, standard deviation, smoothness, skewness, uniformity and entropy were calculated form the histogram of the SHG and TPEF intensity distributions. For a histogram with *L* possible intensity levels, we used *z* to represent a random variable indicating intensity and *p(z)* to represent the probability of the intensity levels in that region. Therefore, the six histogram-based texture features could be calculated as:

,

,
,

,

,
.

- 1. *GLCM-based texture analysis*

To extract the information regarding the relative position of pixels with respect to each other, GLCM-based analysis was implemented. For an image with *K* gray levels, the GLCM was a measurement of the probability *p(i, j)* from a pair of pixels with gray level *i* and *j*, which were separated from each other by the displacement vector *d*. The four GLCM-based texture features were calculated with a *d* of pixels at 1, 2, 3, 4 and four different directions at 0, 45, 90 and 135 degrees. For calculated GLCM-based features, the results obtained from different distances and directions were averaged to get the final outputs. These four GLCM-based texture features could be calculated as:

$Contrast=\sum_{i=1}^{K} \sum_{j=1}^{K} (i-j)^{2}p(i,j)$,

$Correlation=\sum_{i=1}^{K} \sum_{j=1}^{K} \frac{(i-m_{r})(j-m_{c})p(i,j)}{\sigma_{r}\sigma_{c}}$,

$Energy=\sum_{i=1}^{K} \sum_{j=1}^{K} p(i,j)^{2}$,
$Homogeneity=\sum_{i=1}^{K} \sum_{j=1}^{K} \frac{p(i,j)}{1+|i-j|}$.

**Table S1 List of features**

| **Type** | **Feature name** | **Description** |
| --- | --- | --- |
| **Morphology** | **Alignment** | Describe the order degree of fiber-like structure. A larger value corresponds to a more orderly fibrous structure. |
|  | **Density** | Describe the proportion of fiber-like structure in space. |
|  | **Width** | Diameter of fiber-like structure. |
|  | **Length** | Length of fiber-like structure. |
| **Histogram** | **Mean density** | Average intensity of multiphoton signal. |
|  | **Standard deviation** | Average contrast of multiphoton signal. |
|  | **Smoothness** | Intensity consistency in a region. The value 0 is for an image with constant intensity. |
|  | **Skewness** | The value 0 is for symmetric histograms; positive for histograms skewed to the right of the mean; and negative for histograms skewed to the left. |
|  | **Uniformity** | The value is maximum when all intensity values are equal. |
|  | **Entropy** | A measure of randomness. |
| **GLCM** | **Contrast** | Measuring the intensity contrast between a pixel and its neighbors over the entire image. |
|  | **Correlation** | Measuring how a pixel is correlated to its neighbors over the entire image. |
|  | **Energy** | The sum of squared elements in M. |
|  | **Homogeneity** | Measuring the closeness of the distribution of elements in the M to the diagonal of M. |

*Note: M stands for co-occurrence matrix*

**2. Supplementary Figures**


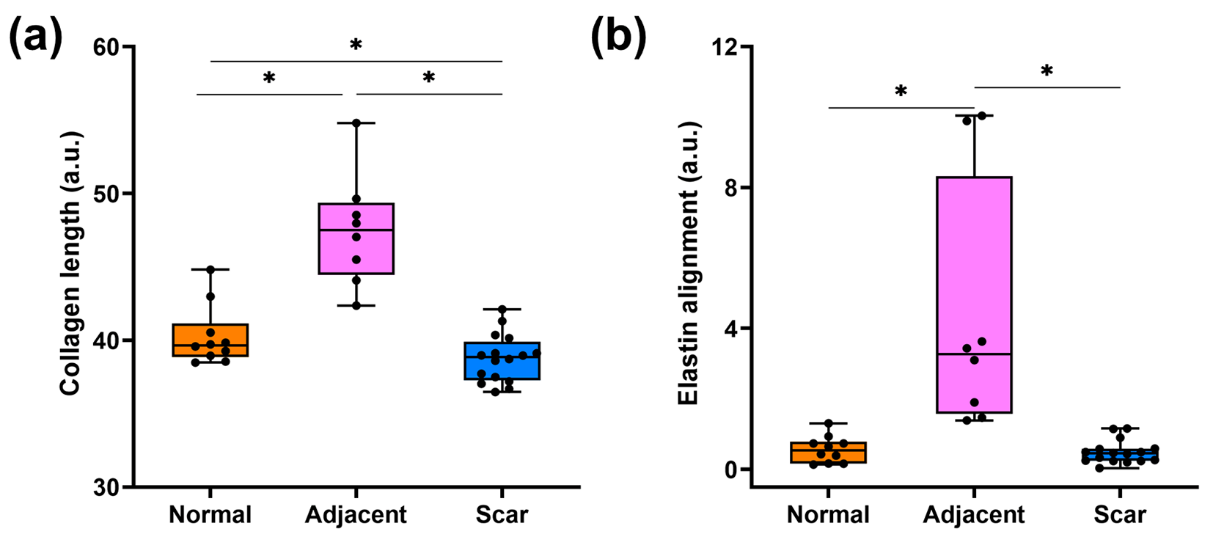


Fig. S1 Representative morphological features. Boxplot of collagen length (a) and elastin alignment (b) for normal, adjacent and scar tissues. *, *p* < 0.05.


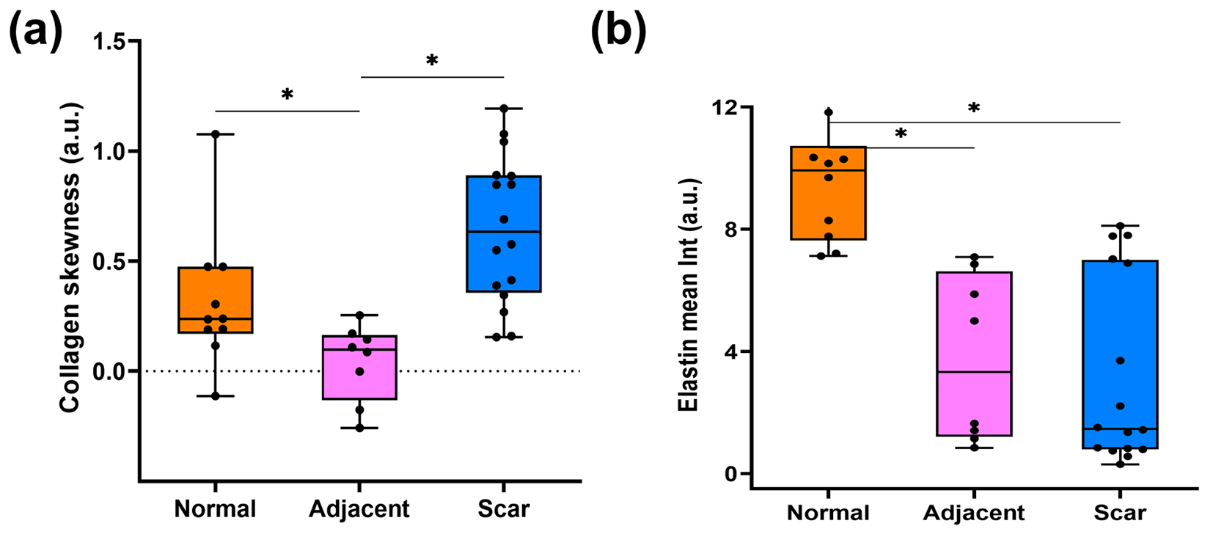


Fig. S2 Representative histogram-based features. Boxplot of collagen skewness (a) and average intensity of elastin (b) for normal, adjacent and scar tissues. *, *p* < 0.05.


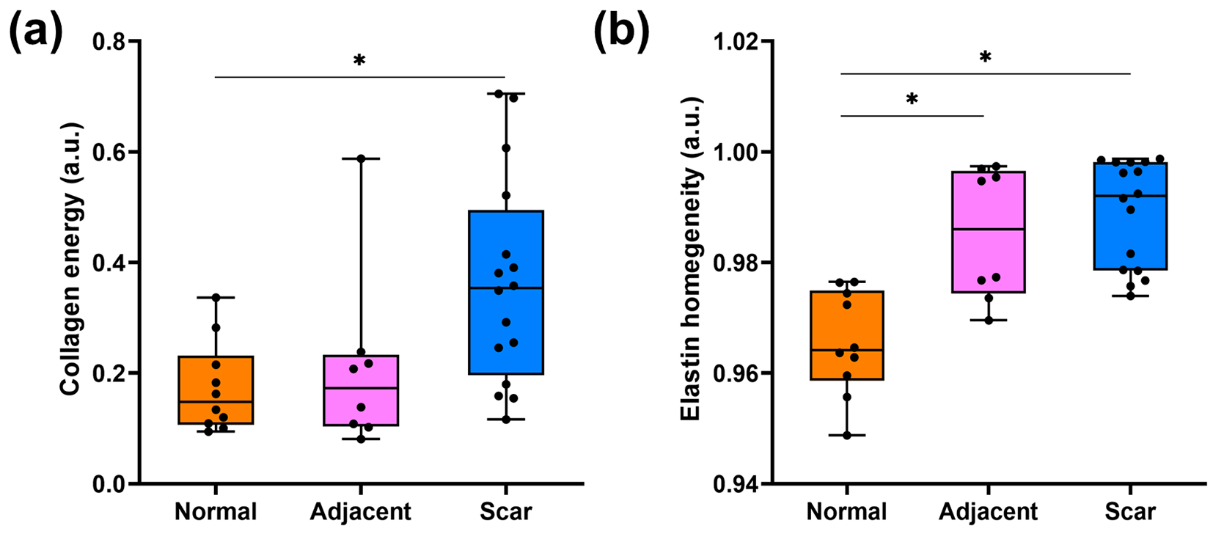


Fig. S3 Representative GLCM-based features. Boxplot of collagen energy (a) and elastin homogeneity (b) for normal, adjacent and scar tissues. *, *p* < 0.05.
